# The metabolic intersection between immunosenescence and neuroinflammation in amyotrophic lateral sclerosis

**DOI:** 10.1186/s12950-025-00460-y

**Published:** 2025-08-27

**Authors:** Victoria S. K. Tsang, Andrea Malaspina, Sian M. Henson

**Affiliations:** 1 Translational Medicine and Therapeutics, William Harvey Research Institute, Barts and The London School of Medicine & Dentistry, Queen Mary University of London, London, United Kingdom; 2 Queen Square Motor Neuron Disease Centre, Neuromuscular Department, Institute of Neurology, University College London, London, United Kingdom; 3 Neuroscience and Trauma Centre, Blizard Institute, Barts and The London School of Medicine & Dentistry, Queen Mary University of London, London, United Kingdom

**Keywords:** Immunosenescence, Neuroinflammation, T cells, Microglia, Amyotrophic lateral sclerosis

## Abstract

Amyotrophic lateral sclerosis (ALS) is a neurodegenerative disease characterized by the progressive loss of motor neurons. Although it is traditionally viewed as a neuron-centric disease, neurodegeneration is increasingly linked to immunosenescence and age-related immune dysfunction, but the mechanisms connecting immune ageing to neurodegeneration remain poorly understood. In this review, we explore how metabolic reprogramming, especially the loss of metabolic plasticity in senescent immune cells, drives neuroinflammation in ALS. Senescent immune cells, including microglia and T cells, exhibit mitochondrial dysfunction, redox imbalance, impaired autophagy, and altered nutrient-sensing pathways that impair their homeostatic and reparative capacities. These cells adopt a metabolically demanding pro-inflammatory phenotype, sustaining an inflammatory secretome while promoting glial activation and neuronal damage. Finally, we discuss how targeting immunometabolic pathways may offer new therapeutic opportunities to restore immune balance, mitigate neuroinflammation, and potentially slow ALS progression. Understanding the metabolic basis of immune ageing is essential for developing effective, age-tailored interventions for ALS.

## Introduction

Amyotrophic lateral sclerosis (ALS) is a progressive neurodegenerative disorder characterized by the degeneration of upper and lower motor neurons [1–3]. The disease typically progresses rapidly, presenting with muscle weakness, atrophy, and paralysis, ultimately resulting in respiratory failure [1, 4]. Despite decades of research, the mechanisms underlying ALS remain incompletely understood. ALS is phenotypically and clinically heterogeneous, with variable sites of onset and rates of disease progression. Recent advances in biomarker development, such as the measurement of neurofilaments in biofluids, have enabled the stratification of individuals with ALS based on disease progression rates [5]. However, an effective cure remains elusive. The median survival is on average 3–5 years from the onset of symptoms, and only 10–20% of individuals with ALS survive longer than 10 years [6, 7]. While ALS has traditionally been framed as a primarily neuron-centric disease, there is increasing evidence that non-cell-autonomous pathological processes play essential roles in modulating disease progression [8].

Age is the most significant risk factor for ALS [9]. Only approximately 5–10% of ALS cases are familial (fALS), associated with mutations in genes such as *SOD1*, *TARDP*, *FUS*, and *C9orf72* [2, 10, 11]. Most cases are sporadic (sALS) and are caused by a complex interplay of genetic predispositions and environmental exposures with no evident family history of ALS [10]. The incidence of sALS increases markedly with age, yet the underlying biological mechanisms by which ageing contributes to ALS pathogenesis remain poorly understood.

Emerging evidence suggests that immunosenescence may play a pivotal role in the neuroinflammatory processes characteristic of ALS [12–14]. One of the hallmarks of ageing, immunosenescence, refers to the gradual decline in immune competence [15, 16]. Immune dysfunction in ALS is multifaceted, featuring elements of hyperinflammation, autoimmunity, and impaired neuroprotective responses. This includes chronic low-grade systemic inflammation, referred to as inflammageing, inappropriate activation of innate and adaptive immune responses, and an imbalance in regulatory control mechanisms [12].

Senescent immune cells reprogram their metabolism and shift toward a pro-inflammatory phenotype, contributing to sustained neuroinflammation and neurodegeneration [12, 17, 18]. The key immune alterations observed in ALS include reduced regulatory T cells (Tregs), expansion of senescent CD8^+^ terminally differentiated effector memory (T_EMRA_) cells, and persistent activation of microglia and astrocytes within the central nervous system (CNS) [19, 20]. These changes contribute to a toxic inflammatory environment driven by dysregulated immune surveillance and heightened oxidative stress. Increased circulating pro-inflammatory cytokines, reactive oxygen species (ROS), and abnormal complement signalling reflect systemic immune imbalance. These immune alterations not only accelerate neuronal vulnerability but also mirror features of immunosenescence, suggesting that ALS involves an accelerated or maladaptive form of immune ageing [21].

Understanding how ageing shapes immune function and metabolism, both systemically and within the CNS, is crucial for revealing the pathophysiology of ALS. This review aims to synthesize emerging evidence linking immunometabolic dysregulation to immunosenescence and neuroinflammation in ALS. We explored how metabolically impaired, or senescent, microglia and T cell populations may mediate disease progression. Framing ALS within the context of immune ageing and metabolic dysfunction may reveal novel directions for research and potential treatment strategies to target the progression of the disease.

## Neuroinflammation plays a pivotal role in ALS pathogenesis

Neuroinflammation is increasingly recognized as a central driver in the pathogenesis of ALS. It refers to the immune response mounted by glial cells, particularly microglia and astrocytes, in response to stimuli such as trauma, infection, or neurodegeneration [19, 20]. While not unique to ALS, neuroinflammation is a hallmark shared by several neurodegenerative disorders, including Alzheimer’s disease, Parkinson’s disease, and multiple sclerosis [22–24]. In ALS, neuroinflammation contributes predominantly to disease progression.

**Fig. 1:**
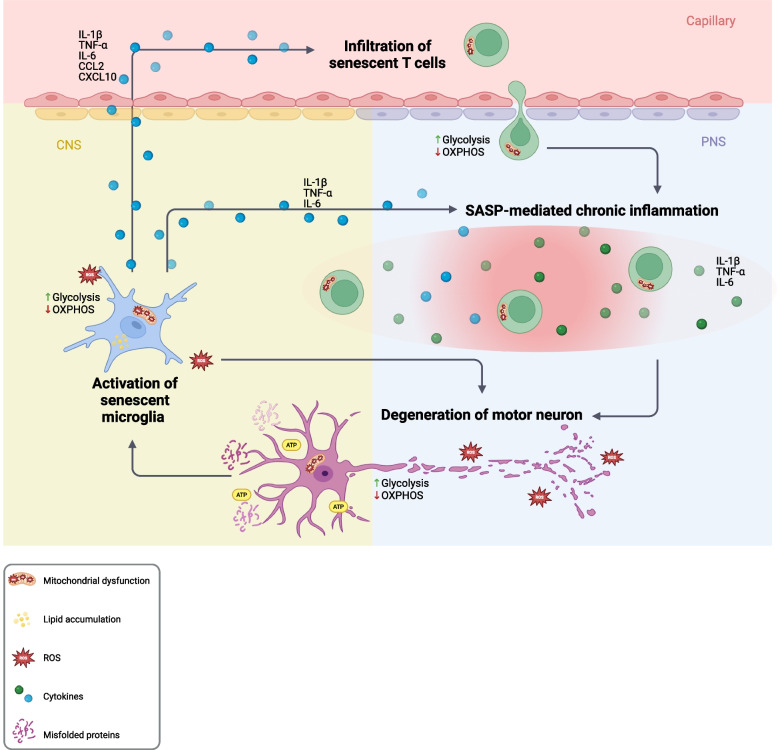
Schematic diagram of neuroinflammation in ALS driven by senescent cells (created using BioRender). In ALS, metabolic dysfunction in motor neurons is characterized by mitochondrial impairment, increased reactive oxygen species (ROS), elevated glycolysis, and reduced oxidative phosphorylation. These metabolic disturbances lead to neuronal injury and the release of danger-associated molecular patterns (DAMPs), including extracellular ATP and aggregated TDP-43 or SOD1, which activate microglia. Senescent microglia mirror these metabolic impairments, as well as lipid droplet accumulation and a diminished capacity to clear toxic protein aggregates, thereby amplifying neurodegeneration. In addition, they secrete a pro-inflammatory senescence-associated secretory phenotype (SASP) rich in cytokines and chemokines, which sustains chronic neuroinflammation and accelerates motor neuron loss. This inflammatory milieu also promotes the recruitment and infiltration of senescent T cells with impaired proliferative and regulatory functions, further reinforcing the SASP and perpetuating a self-sustaining cycle of cellular stress, immune dysfunction, and neuronal injury

Neuronal injury in ALS leads to the release of misfolded proteins, including superoxide dismutase 1 (SOD1) and TAR DNA-binding protein 43 (TDP-43) [25, 26]. The nuclear depletion and cytoplasmic mislocalization of TDP-43 result in the inclusion of cryptic exons in RNA splicing and the production of aberrant cryptic peptides, which have been detected in the CSF of individuals with ALS [27–29]. These non-canonical peptides may be presented on MHC class I molecules by stressed or damaged neurons, particularly under conditions of oxidative stress or neuroinflammation, potentially activating cytotoxic CD8^+^ T cells and contributing to selective neuronal injury [30]. While intrinsic stressors such as SOD1 aggregation can initiate neurotoxicity, this process is amplified by non-neuronal cells, especially glia (Fig. 1) [31]. Neuronal protein aggregates are taken up by glial cells and sensed by pattern recognition receptors (PRRs), such as CD14, TLR2, TLR4, and scavenger receptors [32, 33]. This innate immune activation leads to glial reactivity and triggers a pro-inflammatory cascade, including the secretion of cytokines such as tumour necrosis factor alpha (TNF-α) and interleukin-1 beta (IL-1β) and the production of ROS and reactive nitrogen species (RNS) [34]. These mediators exacerbate motor neuron injury, intensifying local inflammation and creating a self-perpetuating cycle of neurodegeneration. Chronic activation of microglia and astrocytes disrupts synaptic plasticity and neuronal homeostasis. The release of cytokines such as TNF-α, CCL5 and CXCL10, as well as damage-associated molecular patterns (DAMPs), further recruits immune cells to the CNS, amplifying the inflammatory milieu [35]. Mouse models of ALS and postmortem human studies have consistently shown glial activation, further supporting its pathological role in immune dysregulation [23, 36–38].

In addition to neural and glial dysfunction, ALS is characterized by systemic abnormalities in immune regulation. Aberrant immune responses target CNS components and involve both the innate and adaptive arms of the immune system [39]. This is evidenced by peripheral immune cell infiltration, increased pro-inflammatory cytokine levels, and increased expression of PRRs on activated glial cells [40]. Peripheral immune alterations include significant shifts in T cell populations [41]. As mentioned previously, the reduced Tregs and expansion of senescent CD8^+^ T_EMRA_ cells promote a pro-inflammatory environment and oxidative stress [19, 20]. It has been shown that lower Treg levels correlate with faster disease progression, underscoring their likely role in tempering neuroinflammation [19]. Moreover, T cell responses in both peripheral blood and cerebrospinal fluid (CSF) have been identified as predictive markers of disease progression [42]. Peripheral inflammation is further evidenced by elevated levels of circulating monocytes, lymphocytes, and cytokines such as IL-6, IL-8, and TNF-α. Increased fibroblast growth factor (FGF) levels and systemic inflammatory markers have also been associated with an increased risk of ALS [43], implicating peripheral immune dysregulation as a potential contributor to central neuroinflammation.

Together, these findings reveal a complex and multifactorial inflammatory landscape in ALS, where CNS-resident glial activation, peripheral immune cell infiltration, and immune ageing converge to drive neurodegeneration. These immune shifts mirror age-related immunosenescence, suggesting that ALS pathophysiology may reflect an accelerated or dysregulated ageing process within the immune system.

## Immunosenescence in neuroinflammation in ALS

Neuroinflammation in ALS is intensified by age-related immune changes, particularly inflammageing and cellular senescence [24, 39, 44]. Although immunosenescence, the progressive deterioration of immune competence with age, is a well-recognized contributor to chronic inflammation, its role in ALS has only recently gained attention [39].

A central mechanism driving immunosenescence is inflammageing, driven by the accumulation of senescent cells [45–47]. Immune deterioration is characterized by thymic involution, a decline in naïve T cell production, expansion of senescent T cell subsets, and impaired immune surveillance [43, 48–50]. Yildiz et al. previously demonstrated that the systemic elevation of senescent and late-memory T and B lymphocytes in the peripheral blood was more pronounced in individuals with faster disease progression and bulbar-onset ALS [13]. The study also revealed that higher frequencies of CD4^+^ central memory T cells and CD95^+^ CD8^+^ T cells were associated with shorter survival and were correlated with worse functional status at baseline. These findings suggest that the accumulation of senescent and late-memory lymphocytes contributes to the chronic inflammation observed in ALS.

Senescent cells also adopt a pro-inflammatory phenotype and secrete a mixture of inflammatory mediators, including IL-6, TNF-α, and IL-1β, known as the senescence-associated secretory phenotype (SASP) (Fig. 1) [51]. The SASP disrupts tissue homeostasis, activates microglia and astrocytes, and perpetuates neuroinflammation, forming a vicious cycle of cellular dysfunction and immune activation in the CNS [21, 47, 52, 53]. Multiple studies have shown that increased concentrations of pro-inflammatory cytokines are associated with the SASP in the blood and CSF of individuals with ALS [51, 54–57]. These changes weaken immune defence mechanisms while simultaneously amplifying the neurodegenerative cascade.

Senescent cells not only sustain inflammation through the SASP but also propagate senescence systemically by altering their microenvironment and impairing the function of other immune cells [13]. With advancing age, the accumulation of genomic damage gives rise to DAMPs, which activate type I interferon (IFN) responses and inflammasome signalling pathways. In glial cells, this inflammatory activation is often mediated via the NLRP2 inflammasome, contributing to sustained neuroinflammation [21]. Senescent glial cells, particularly microglia and oligodendrocyte precursor cells, exhibit diminished regenerative capacity and adopt a pro-inflammatory state, further amplifying the chronic inflammatory environment in the ageing brain.

Importantly, many hallmarks of cellular senescence overlap with the molecular changes observed in the ALS-affected CNS. These include impaired autophagy, disrupted proteostasis, dysregulated RNA splicing, nuclear‒cytoplasmic transport defects, and mitochondrial dysfunction characterized by fragmentation and reduced bioenergetic efficiency [9, 53, 58, 59]. The toxicity of senescent cells is intensified by metabolic stress, including altered glycolysis and reduced ATP production. Given the high energy demands and limited metabolic flexibility of motor neurons, these deficits in cellular energetics likely contribute significantly to neuronal vulnerability and disease progression in individuals with ALS [60, 61].

## Immunometabolism of senescent cells

Immunometabolism, the dynamic relationship between cellular metabolism and immune function, is increasingly recognized as a critical factor in the pathogenesis of ALS [52, 53, 62–66]. Immune cells typically demonstrate metabolic plasticity, switching between glycolysis, oxidative phosphorylation (OXPHOS), the tricarboxylic acid (TCA) cycle, fatty acid oxidation (FAO), the pentose phosphate pathway, and amino acid metabolism depending on their activation status and functional needs [62, 63]. For example, effector T cells rely on glycolysis to support rapid proliferation and cytokine production, whereas Tregs rely more on OXPHOS and FAO.

At the systemic level, most individuals with ALS exhibit hypermetabolism and progressive weight loss, although the exact origins and cell-type-specific contributions remain unclear [67]. Indeed, metabolic flexibility is disrupted, especially in chronically activated or senescent immune cells [34, 68]. These cells maintain a persistent inflammatory phenotype characterized by increased expression of SASP-associated cytokines and increased glycolysis and mitochondrial respiration [52]. This shift is partly sustained by disrupted nicotinamide adenine dinucleotide (NAD) metabolism, particularly via the HMGA-NAMPT axis and overactivation of CD38, leading to NAD +/NADH balance disruption, impaired cellular repair, and sustained inflammatory responses [69].

Additional drivers of this inflammatory metabolic state include inflammasome activation and IL-1 signalling, triggered by age-related accumulation of DAMPs and high glucose levels [70]. This milieu promotes ROS generation, mitochondrial dysfunction, and the activation of stress pathways, which further impair autophagy, reduce metabolic efficiency, and perpetuate neuroinflammation. Dysregulation of nutrient-sensing pathways (mTOR, AMPK, and sirtuins) and reduced autophagy compromise the ability of immune cells to regulate metabolic stress and effectively resolve inflammation. In ageing tissues, chronic FAO and incomplete TCA cycle activity generate toxic byproducts such as ketone bodies, exacerbating oxidative stress and cellular damage [68].

Metabolite imbalance is also a hallmark of cellular senescence, often driven by mitochondrial dysfunction, which can alter T cell behaviour and promote a pro-inflammatory phenotype [71]. We propose that metabolic imbalance serves as a pathological link between ALS, immune senescence, and chronic inflammation. The high energy demands of maintaining the SASP in senescent cells drive a shift from basal to highly active metabolic modes, particularly glycolysis. While this supports short-term survival in an inflammatory microenvironment, it comes at the expense of key immune functions [72]. T cells, for example, show reduced proliferative and migratory capacity despite maintaining cytokine output [73].

In addition to their role in energy production, metabolic intermediates such as citrate and α-ketoglutarate (αKG) also influence gene expression and protein synthesis, including through mechanisms of translational repression that can shape the senescent microenvironment [74]. Mitochondrial integrity is particularly critical for the suppressive activity of Tregs, and αKG plays a key role in maintaining Treg identity by regulating DNA methylation at the *FOXP3* locus [75]. Elevated αKG levels, however, have been shown to inhibit Treg differentiation and enhance the production of inflammatory cytokines. Thus, impaired mitochondrial metabolism may shift the immune balance toward activation and inflammation in ALS. Notably, metabolites are not confined to intracellular compartments; senescent fibroblasts actively secrete citrate, glutamate, aspartate, and lactate, along with lipid mediators, as part of the SASP, contributing to a metabolically and immunologically active extracellular milieu [76, 77].

Overall, mitochondrial dysfunction, impaired glial support, defective protein clearance, and immune-driven oxidative stress disrupt neuronal energy homeostasis, creating a vicious cycle of neurodegeneration in ALS. These systemic immune alterations impact the PNS and CNS, where neuroimmunometabolism modulates neuroinflammation through glial and peripheral immune cell interactions that sustain metabolic stress and neuronal injury [34].

## Cell type-specific metabolic alterations in ALS

### Neurons: vulnerable targets of metabolic and glial dysfunction

Motor neurons are among the most metabolically demanding cell types and rely heavily on oxidative phosphorylation for energy production. This dependence renders them particularly susceptible to mitochondrial dysfunction and oxidative stress [67, 78, 79]. In ALS, both autonomous neuronal defects and non-cell-autonomous changes contribute to motor neuron degeneration. One notable systemic feature is hypermetabolism, which can exacerbate pathological protein expression. For example, metabolic stress upregulates TDP-43, a hallmark of ALS pathology [80]. TDP-43 mislocalizes to mitochondria and binds mitochondria-transcribed messenger RNAs encoding complex I subunits, leading to complex I disassembly and disruption of the respiratory chain [80, 81].

This mitochondrial disruption in ALS is accompanied by broader structural and functional deficits, including impaired glucose uptake and metabolism in the CNS, reduced ATP production, and elevated ROS generation [67, 78]. Induced pluripotent stem cell-derived ALS neurons further demonstrate impaired mitochondrial respiration coupled with compensatory upregulation of glycolysis [79]. A key driver of this mitochondrial dysfunction is the hyperacetylation of mitochondrial proteins resulting from decreased activity of the mitochondrial deacetylase sirtuin-3 [79]. However, while some studies report increased glycolysis, others indicate glycolytic insufficiency, suggesting that these compensatory mechanisms are ultimately inadequate to meet neuronal energy demands [67]. Energy failure also compromises critical cellular functions such as synaptic transmission and axonal transport, both of which are strongly energy dependent.

Metabolic disturbances are also evident in glial support cells, creating a feedback loop of inflammation and neurodegeneration. For example, neurons exhibit glutamate-mediated toxicity and are deprived of essential metabolic support because astrocytes fail to clear glutamate, and oligodendrocytes exhibit impaired lactate transport [67, 82, 83]. Neuronal damage also leads to the release of misfolded and aggregated proteins and ATP, which can activate glial and peripheral immune cells [84–88]. Impaired autophagy in microglia prevents the clearance of toxic protein aggregates such as TDP-43 and SOD1, reinforcing a pro-inflammatory and neurotoxic environment (Fig. 1) [86–88].

### Microglia: dynamic shifts in metabolic and inflammatory states

Microglia, the primary immune surveillance cells in the CNS, undergo profound metabolic reprogramming in ALS. Early in the disease, these cells exhibit a neuroprotective M2-like phenotype characterized by anti-inflammatory cytokine production and effective phagocytosis. As diseases progress, autophagy of abnormal proteins, including SOD1 and TDP-43, aggregates from degenerating neurons, promotes a pro-inflammatory M1-like phenotype and the secretion of pro-inflammatory cytokines such as IL-1β, IL-6, and TNF-α [87–89]. Indeed, microglia isolated from mice with early-stage ALS express relatively high levels of M2 microglia phenotype markers, whereas microglia isolated from end-stage ALS express high levels of pro-inflammatory markers [89]. Microglia also exhibit features of senescence, as shown in SOD1-mutant models [18, 90]. Senescent microglia exhibit impaired autophagy and mitochondrial dysfunction, limiting their phagocytic capacity and enhancing neuroinflammation through elevated ROS and TDP-43 accumulation (Fig. 1) [90, 91]. Moreover, C9orf72 mutations further exacerbate microglial dysfunction by impairing lysosomal trafficking [20].

Upon protein aggregate engulfment, microglia display metabolic irregularities, such as a glycolytic metabolic profile, succinate accumulation, and TCA cycle disruption [92]. This phenotype switch is accompanied by mTOR and HIF-1α activation and GLUT1 upregulation, further promoting the chronic inflammatory state and contributing to neurodegeneration [93, 94]. Complement-mediated signalling (e.g., via C3a and C5a) further promotes glycolysis and pro-inflammatory polarization in microglia [95]. This finding mirrors findings from Alzheimer’s disease models, where amyloid precursor protein (APP)/presenilin 1 (PS1)-overexpressing microglia from mice exhibited disrupted phagocytosis and increased glycolysis [96, 97]. Notably, APP expression is also elevated in ALS, which may imply a pathologic convergence of metabolic stress and immune dysfunction in microglia, amplifying neurodegeneration in ALS.

### T cells: Tregs in decline and T_EMRA_ cells as inflammatory factors

T cells are central to immune surveillance and neuroinflammation regulation in individuals with ALS. Following the blood-brain barrier disruption and glial activation, peripheral T cells infiltrate the CNS to exacerbate neurodegeneration, depending on their subtype and activation state [98, 99]. Upon activation, T cells normally shift from OXPHOS to glycolysis to meet the energy demands of proliferation and cytokine production, but this metabolic flexibility is impaired by immunosenescence in ALS.

Individuals with ALS have reduced numbers of CD4 ^+^ FOXP3 ^+^ Tregs and increased numbers of CD8^+^ T_EMRA_ cells, reflecting an aged and pro-inflammatory immune profile [13, 100]. Both subsets display reduced proliferative capacity, altered cytokine profiles, and increased reliance on dysfunctional metabolic pathways [13]. Senescent CD8 ^+^ T_EMRA_ cells rely heavily on glycolysis but exhibit low mitochondrial membrane potential. CD4 ^+^ T_EMRA_ cells have increased mitochondrial mass but suffer from oxidative damage and inefficient respiration due to diminished NAD ^+^ availability. Reduced NAD ^+^ levels impair mitochondrial function, diminishing antigen responsiveness and increasing susceptibility to apoptosis [13]. In addition to the downregulation of FoxP3 expression in rapidly progressing ALS, Tregs exhibit impairments in FAO and mitochondrial metabolism, compromising Treg-mediated immune and suppressive regulation [100, 101]. This loss of regulatory control allows effector T cells to proliferate unchecked, promotes microglial activation, and amplifies the neuroinflammatory milieu (Fig. 1) [100].

These metabolic imbalances are heightened by dysregulated mTOR signalling, which further impairs T cell proliferation, survival, and effector function. Complement components such as C3a and C5a exacerbate this dysfunction by shifting T cells toward a glycolytic, pro-inflammatory phenotype [95]. These maladaptive shifts contribute to the production of ROS, fuelling neuroinflammation and increasing neuronal vulnerability. Collectively, the metabolic alterations in T cells reflect systemic immunosenescence and underscore the loss of homeostatic immune control in ALS.

## Therapeutic implications

Despite recent progress, most notably with antisense oligonucleotides such as tofersen, which effectively reduce the synthesis of the SOD1 protein, no therapy capable of halting or reversing ALS progression has been developed. Immunomodulatory approaches, such as ex vivo–expanded autologous Treg infusions to suppress the proliferation of cytotoxic T cells and the activation of microglia, have shown transient benefits, but disease progression resumes after treatment terminates [102]. This limited durability may reflect the highly pro-inflammatory and senescence-promoting environment of ALS, which impairs the expansion and suppressive function of infused Tregs and may promote their senescent-like phenotype [19].

More recently, the MIROCALS study investigated the efficacy and safety of low-dose IL-2 as an adjunct to riluzole in people living with ALS [103]. While the overall survival benefit did not reach statistical significance, a significant improvement in survival was observed in individuals with low-to-medium CSF levels of phosphorylated neurofilament heavy chain. These findings suggest the potential for precision medicine approaches informed by biomarker stratification. Although low-dose IL-2 is promising because of its Treg-enhancing effects, its efficacy is likely constrained by the underlying immune dysfunction in ALS, which is driven by immunosenescence, metabolic dysregulation, and chronic inflammation.

To address these barriers, emerging therapeutic strategies are now focused on the root causes of immune dysfunction. Interventions aimed at restoring immune cell metabolism, including the use of mTOR inhibitors, NAD^+^ precursors, and mitochondrion-targeted antioxidants, may help enhance the function and resilience of regulatory immune cells [104]. Additionally, senotherapeutics, such as senolytics (e.g., dasatinib and quercetin) and senomorphics targeting the SASP, have shown neuroprotective effects in preclinical models of neurodegeneration [105, 106].

Ultimately, the limited efficacy of prior immunotherapies in ALS may reflect a failure to consider the hostile neurodegenerative microenvironment. A rational next step could involve combining low-dose IL-2 with senolytics or metabolic modulators to synergistically enhance immune regulation and the durability of the response in individuals with ALS. Future strategies may benefit from the development of engineered Tregs, such as chimeric antigen receptor (CAR) Tregs, which are designed to resist senescence and metabolic exhaustion while targeting key drivers of neuroinflammation.

## Conclusion

Traditionally, ALS has been viewed primarily as a neurodegenerative disorder, with limited attention given to the role of non-neuronal cells. However, recent studies have highlighted chronic inflammation and metabolic dysregulation as central drivers of ALS pathogenesis. Immunosenescence, which is characterized by disrupted immunometabolism and a persistent pro-inflammatory state, contributes to disease progression not only in individuals with ALS but also in those with other neurodegenerative conditions, such as Alzheimer’s disease, Parkinson’s disease, and multiple sclerosis. Emerging evidence reveals a dynamic interplay between peripheral immune dysfunction and CNS glial responses, where each compartment reinforces the other. This bidirectional crosstalk creates a self-perpetuating loop; mitochondrial dysfunction promotes glial activation, which in turn sustains inflammation and accelerates neurodegeneration. A deeper understanding of the metabolic pathways underlying cell-specific dysfunctions is crucial. Such insights could guide the development of targeted therapeutic strategies aimed at correcting immune dysregulation and disrupting the inflammatory cycle, ultimately slowing ALS progression.

## Data Availability

No datasets were generated or analysed during the current study.

## Abbreviations

ALS: Amyotrophic lateral sclerosis
APP: Amyloid precursor protein
CAR: Chimeric antigen receptor
CNS: Central nervous system
CSF: Cerebrospinal fluid
DAMPs: Damage-associated molecular patterns
fALS: Familial ALS
FAO: Fatty acid oxidation
FGF: Fibroblast growth factor
IFN: Interferon
IL-6: Interleukin-6
NAD: Nicotinamide adenine dinucleotide
OXPHOS: Oxidative phosphorylation
PRRs: Pattern recognition receptors
PS1: Presenilin 1
ROS: Reactive oxygen species
sALS: Sporadic ALS
SASP: Senescence-associated secretory phenotype
SOD1: Superoxide dismutase 1
TCA: Tricarboxylic acid
TDP-43: TAR DNA-binding protein 43
TEMRA: Terminally differentiated effector memory
TNF-α: Tumour necrosis factor alpha
Tregs: Regulatory T cells
αKG: α-Ketoglutarate
